# Spleen-targeted mRNA delivery via long-chain PEGylated lipids at low molar ratio enhances antitumor immunity against melanoma

**DOI:** 10.1016/j.omtn.2025.102803

**Published:** 2025-12-12

**Authors:** Shiyu Liu, Longlong Zhang, Wenbo Wu, May Yee Chen, Feng Qian

**Affiliations:** 1 School of Pharmaceutical Sciences, Beijing Frontier Research Center for Biological Structure, and Key Laboratory of Bioorganic Phosphorus Chemistry & Chemical Biology (Ministry of Education), Tsinghua University, Beijing, P.R. China

**Keywords:** MT: delivery strategies, mRNA delivery, lipid nanoparticles, PEGylated lipids, spleen targeting, tumor vaccine

## Abstract

Lipid nanoparticles (LNPs) are pivotal for mRNA delivery, yet predominant hepatic accumulation limits their therapeutic application in immunologically active sites like the spleen. Engineering LNPs for splenic mRNA delivery is therefore essential for advancing mRNA-based immunotherapies. Here, we rationally designed a series of LNPs by modulating the alkyl chain length of PEGylated lipids (fast-shedding C14 DMG-PEG vs. slow-shedding C18 DSG-PEG) and their molar ratios (0.75% and 1.5%). This systematic variation precisely controlled nanoparticle physicochemical properties and protein corona composition. Among these formulations, DSG-PEG LNPs at 0.75% mol exhibited the largest size (∼170 nm by DLS; ∼60 nm core by cryo-EM) and a distinct protein corona. Notably, this DSG-PEG 0.75% mol formulation demonstrated significantly enhanced selective spleen accumulation and efficient mRNA delivery *in vivo*. Furthermore, the optimized LNP potently activated dendritic cells, expanded antigen-specific CD4^+^ T cell populations, and significantly inhibited tumor growth in a B16-ovalbumin (OVA) melanoma model. Collectively, our results establish a rational strategy via precise PEGylated lipid engineering to redirect LNP biodistribution from the liver to the spleen. These findings validate the spleen as a critical target for mRNA vaccines and provide a versatile platform for next-generation cancer immunotherapies.

## Graphical abstract

**Figure:**
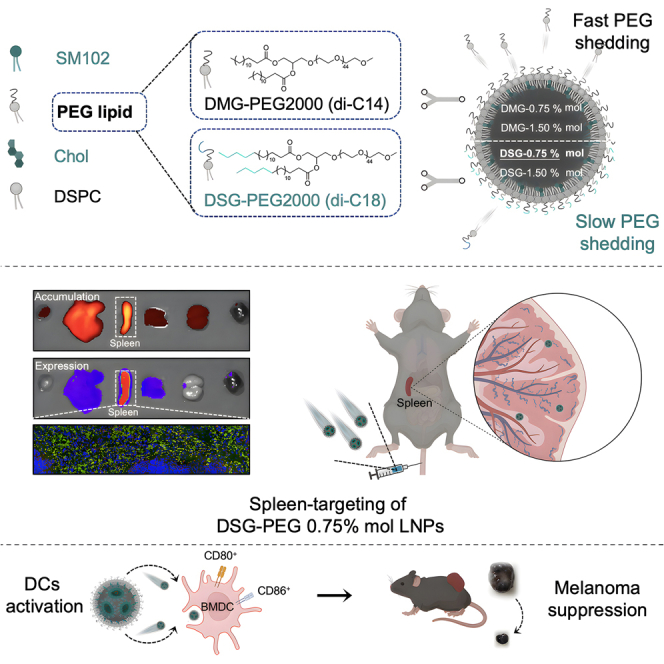

## Teaser

Long-chain PEGylated lipids at low molar ratios reprogram lipid nanoparticle biodistribution from the liver to the spleen. This spleen-targeted formulation efficiently delivers mRNA to dendritic cells and elicits strong antitumor immunity, offering a simple and scalable strategy to enhance the efficacy of mRNA-based cancer immunotherapies.

## Introduction

Messenger RNA (mRNA) vaccines have emerged as highly promising tools for cancer immunotherapy due to their ability to rapidly encode tumor-specific antigens and stimulate potent antigen-specific immune responses.^1^^,^^2^^,^^3^ Clinical studies combining personalized mRNA vaccines with immune checkpoint inhibitors have already demonstrated significant therapeutic benefits in melanoma, underscoring their potential in adjuvant cancer therapy.^4^^,^^5^ However, clinical translation remains challenged by mRNA instability and insufficient delivery to immune-active tissues like the spleen, for effective immune priming.^6^^,^^7^

Lipid nanoparticles (LNPs) are the most widely used delivery systems for systemic mRNA administration due to their high mRNA encapsulation efficiency, low immunogenicity, and favorable safety profile.^8^^,^^9^^,^^10^^,^^11^ However, a key challenge of LNP-based mRNA delivery is their predominant accumulation in the liver, primarily mediated by apolipoprotein E (ApoE) receptors expressed on hepatocytes.^12^^,^^13^ This hepatic bias limits effective mRNA delivery to immune-active sites such as the spleen, which plays a crucial role in systemic antigen presentation and T cell priming.^14^ As a secondary lymphoid organ enriched with dendritic cells (DCs) and macrophages, the spleen represents an attractive but underexploited target for mRNA vaccine delivery.^15^^,^^16^^,^^17^^,^^18^^,^^19^ Therefore, engineering LNPs for selective spleen accumulation holds significant promise as an ideal strategy for enhancing mRNA vaccines efficacy by harnessing the spleen’s immunological functions.

As a crucial component of LNP, the PEGylated lipids are incorporated into the formulation to form a hydrated layer, providing colloidal stability and preventing fusion. Meanwhile, the PEG layer offers a “stealth” effect that prolongs circulation time by evading uptake by the reticuloendothelial system.^20^^,^^21^ Furthermore, the surface density and molecular structure of PEGylated lipids, especially the alkyl chain length of PEGylated lipid, significantly modulate key physicochemical properties of LNPs, such as particle size and surface charge, influence the formation of the protein corona, and thus collectively affect biodistribution profiles.^22^^,^^23^^,^^24^^,^^25^

Given these relationships, rational modulation of PEGylated lipid composition emerges as a promising strategy to reduce hepatic retention and enhance delivery of LNPs to immunologically active tissues. However, the precise mechanisms underlying biodistribution and selective organ targeting of LNPs influenced by PEGylated lipid remain incompletely understood. While the intrinsic kinetic and thermodynamic properties of nanoparticles affect their *in vivo* distribution, the physiological characteristics of target organs also play critical roles. In particular, the spleen—with its unique filtration function and abundant phagocytic immune cells—represents an ideal target for mRNA vaccine delivery.^26^^,^^27^ Therefore, our study aims to selectively target the spleen to enhance the efficacy of mRNA-based tumor immunotherapy by LNPs.

To overcome the conventional liver-biased biodistribution of LNPs, we systematically engineered a series of mRNA-loaded LNPs formulations by varying both the PEGylated lipid alkyl chain length (short-chain DMG-PEG versus long-chain DSG-PEG) and their molar ratios (0.75% versus 1.5%). The 1.5% PEG-lipid ratio was chosen as a representative “conventional” formulation commonly employed in liver-targeted LNPs, whereas 0.75% represents a reduced-PEG condition that allows assessment of how sparser PEG coverage modulates protein adsorption and organ targeting. This rational design allowed us to precisely manipulate nanoparticle physicochemical properties—including particle size, surface charge, and protein corona profile—parameters critically associated with their biodistribution and therapeutic performance.

Remarkably, our optimized formulation, incorporating long-chain DSG-PEG at a low molar ratio (0.75%), demonstrated selective spleen accumulation with minimal hepatic uptake. This targeted delivery strategy significantly enhanced mRNA expression within splenic DCs, resulting in profound activation of antigen-specific CD4^+^ and CD8^+^ T cell responses. In a clinically relevant B16-OVA melanoma model, our spleen-targeted LNPs elicited robust antitumor immunity, characterized by retarded tumor growth, reduced tumor burden, and enhanced infiltration of cytotoxic T cells expressing granzyme B and IFN-γ.

Together, these findings highlighted the critical influence of PEGylated lipid composition on the biodistribution and immunological efficacy of LNPs, and demonstrate that minor yet tailored alterations in PEGylated lipid structure and molar density can effectively redirect LNPs fate toward the spleen. This tailored design formulation strategy provides a highly scalable and effective LNP platform for enhancing the therapeutic potential of next-generation mRNA-based cancer vaccines.

## Results

### Physicochemical characterization of mRNA-LNPs contained different C-chain length and molar ratio of PEGylated lipid

**Figure 1:**
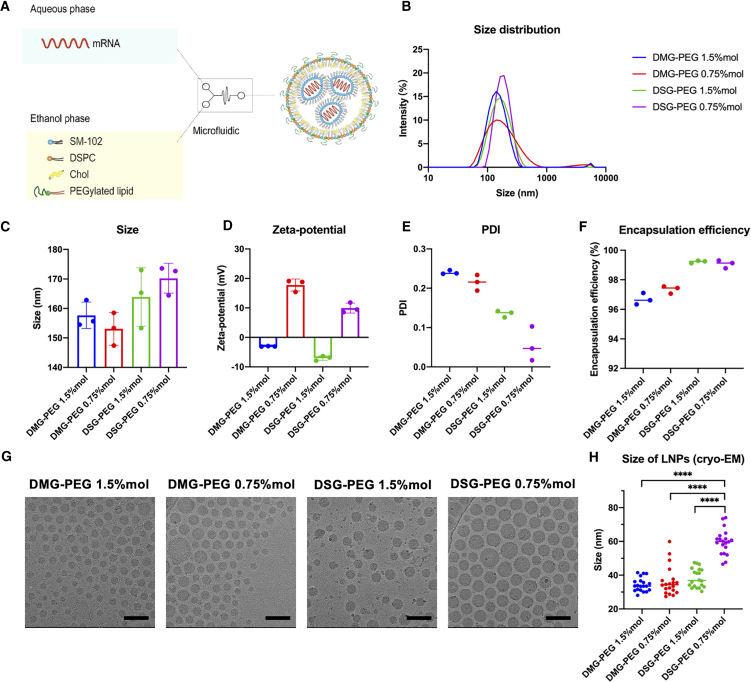
Physicochemical characterization of mRNA-loaded LNPs (A) Microfluidic synthesis of LNPs for mRNA delivery, showing aqueous mRNA phase combining with lipid components dissolved in ethanol (SM-102, DSPC, Chol, PEGylated lipid). (B–F) Physicochemical characterization: (B) Size distribution curves by DLS; (C) Average particle sizes from DLS measurements (nm); (D) Surface zeta potentials (mV); Polydispersity index (PDI) indicating particle uniformity; (F) Encapsulation efficiency (EE %) of mRNA in LNPs. (G) Representative cryo-EM images confirming spherical morphology. Scale bars: 100 nm. (H) Statistical comparison of particle sizes measured by cryo-EM. Data represent means ± SD, ∗∗∗∗*p* < 0.0001.

LNPs were synthesized via a rapid nanoprecipitation method using a microfluidic mixing (Figure 1A). To assess the impact of PEGylated lipid type and molar ratio on LNPs physicochemical characteristics four distinct formulations were systematically evaluated. DLS confirmed uniform particle sizes across all formulations, with polydispersity index (PDI) consistently below 0.2 (Figures 1B and 1E). Specifically, the average diameters for DMG-PEG and DSG-PEG LNPs at a 1.5% molar ratio were approximately 157 nm and 164 nm, respectively. At the lower PEGylated lipid ratio (0.75%), DMG-PEG LNPs measured around 153 nm, whereas DSG-PEG LNPs was slightly larger, around 170 nm (Figure 1C). Notably, DMG-PEG-containing LNPs consistently exhibited smaller particle sizes than their DSG-PEG counterparts, likely due to longer-chain PEGylated lipids (e.g., DSG-PEG) possess stronger membrane anchoring and slower desorption rates, which may hinder lipid mobility and rearrangement during nanoparticle self-assembly, resulting in larger particle formation.^24^^,^^28^ In addition, LNPs formulated with a lower molar ratio of PEGylated lipid also exhibited increased particle sizes, consistent with previous reports, possibly due to reduced steric stabilization and insufficient surface coverage during assembly.^29^

Surface zeta potential measurements (Figure 1D) demonstrated a clear PEG dose-dependent effect. LNPs with 1.5% mol PEGylated lipid exhibited slightly negative surface charges in PBS, attributable to partial deprotonation of the ionizable lipid SM-102 at physiological pH (7.4). Reducing the PEGylated lipid ratio to 0.75% mol notably increased the surface zeta potential, with DMG-PEG and DSG-PEG LNPs reaching approximately +18 mV and +10 mV, respectively. This shift toward positive charge results from reduced PEG shielding, leading to greater exposure of protonated SM-102 lipid.

All formulations achieved high encapsulation efficiencies (>90%) for mRNA (Figure 1F). We next characterized the LNPs using cryo-EM (Figure 1G), which confirmed their spherical morphology and allowed direct visualization of particle size. Compared with DLS, which measures the hydrodynamic diameter including the hydration layer, cryo-EM visualizes dehydrated particle cores, providing a more accurate representation of their true physical size. Notably, cryo-EM measurements revealed significantly larger core particle sizes (∼60 nm) for DSG-PEG 0.75% mol LNPs compared to the other three formulations (all <40 nm, Figure 1H).

Collectively, these data indicate that both PEG molar ratio and lipid anchor length synergistically modulate critical physicochemical attributes of LNPs, particularly surface charge, particle size, and structural homogeneity.

### PEGylated lipid composition modulates protein corona profiles on LNPs

To elucidate how PEGylated lipid alkyl chain length and molar ratio influence protein corona formation, LNPs were incubated with mouse plasma and subjected to sucrose gradient ultracentrifugation. Given density differences between LNPs (∼0.9–1.0 g/mL) and plasma (1.025–1.030 g/mL), a 15%–30% sucrose gradient (1.0577–1.1252 g/mL) was employed. After centrifugation, the topmost fraction containing protein corona-bound LNPs was analyzed. SDS-PAGE confirmed enrichment of corona-bound LNPs in the upper fraction, with most free plasma proteins sedimenting to lower layers (Figure S1).

**Figure 2:**
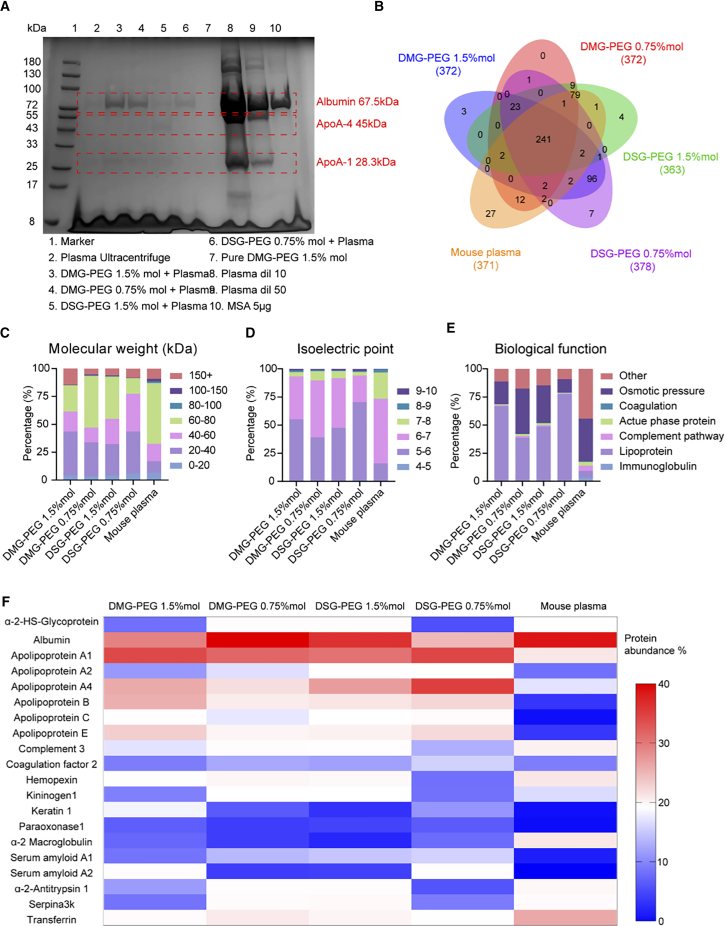
PEGylated lipid composition influenced LNP protein corona profiles (A) SDS-PAGE analysis showing the protein corona profiles after LNPs were incubated with mouse plasma. Lanes 3–6 show protein bands associated with different LNPs formulations, with major corona proteins (Albumin, ApoA-4, ApoA-1) highlighted. (B) Venn diagram indicating the overlap and uniqueness of identified corona proteins across the four LNPs formulations and mouse plasma control. (C–E) Classification of identified corona proteins based on (C) molecular weight, (D) isoelectric point (pI), and (E) biological function. (F) Heatmap showing the relative abundance (%) of the top 20 most enriched proteins in the corona profiles across LNPs types and mouse plasma. The absolute abundance values of these proteins, determined by LC-MS, are listed in Table S2. Corresponding relative abundance percentages used to generate the heatmap are provided in Table S3.

Protein corona profiles obtained via SDS-PAGE exhibited distinct banding patterns across formulations (Figure 2A). DMG-PEG-formulated LNPs showed stronger protein adsorption than DSG-PEG counterparts, as evidenced by more intense protein bands and higher BCA-quantified protein levels, with lower PEG molar ratios further enhancing protein binding across both formulations (Figures 2A and S2).

Proteomic analysis by LC-MS identified approximately 370 distinct proteins. Venn diagram analysis revealed substantial overlap, with 241 shared proteins, yet each formulation contained unique proteins, highlighting distinct corona profiles (Figure 2B). Interestingly, the 20 most abundant proteins represented >90% of total corona mass, indicating that a few proteins dominated corona composition. Predominant corona proteins included albumin (∼67.5 kDa), apolipoprotein A1 (ApoA1, ∼28.3 kDa), and apolipoprotein A4 (ApoA4, ∼45 kDa).

Further classification based on molecular weight (Figure 2C), isoelectric point (Figure 2D), and biological function (Figure 2E) revealed distinct corona enrichment patterns. LNPs coronas contained predominantly smaller proteins (20–40 kDa), especially pronounced in DSG-PEG 0.75% mol formulations (∼75% < 60 kDa). Acidic proteins (pI < 7) dominated all coronas, likely reflecting electrostatic interactions with protonated SM-102. Despite this, surface zeta potential alone did not directly correlate with corona formation, implying involvement of additional factors.

To further evaluate how PEGylated lipid composition affects protein corona formation, we compared the top 20 most abundant proteins identified across LNPs formulations and mouse plasma (Figure 2F). The heatmap clearly shows that DSG-PEG-formulated LNPs exhibited markedly different corona profiles compared to DMG-PEG counterparts, supporting the notion that both PEG type and molar ratio play a key role in corona composition. These findings highlight the potential of PEGylated lipid engineering to fine-tune protein corona signatures and, consequently, modulate the *in vivo* fate of LNPs.

### Long-chain PEGylated lipid LNPs at low molar ratio (DSG-PEG 0.75% mol) enhanced spleen accumulation and mRNA delivery *in vivo*

As previously described, adjusting PEGylated lipid alkyl chain length and molar ratio significantly influenced both LNPs size and protein corona formation. Given the pivotal role these factors play in nanoparticle biodistribution, we further explored the *in vivo* distribution and mRNA delivery efficiency of the four LNPs formulations developed.

**Figure 3:**
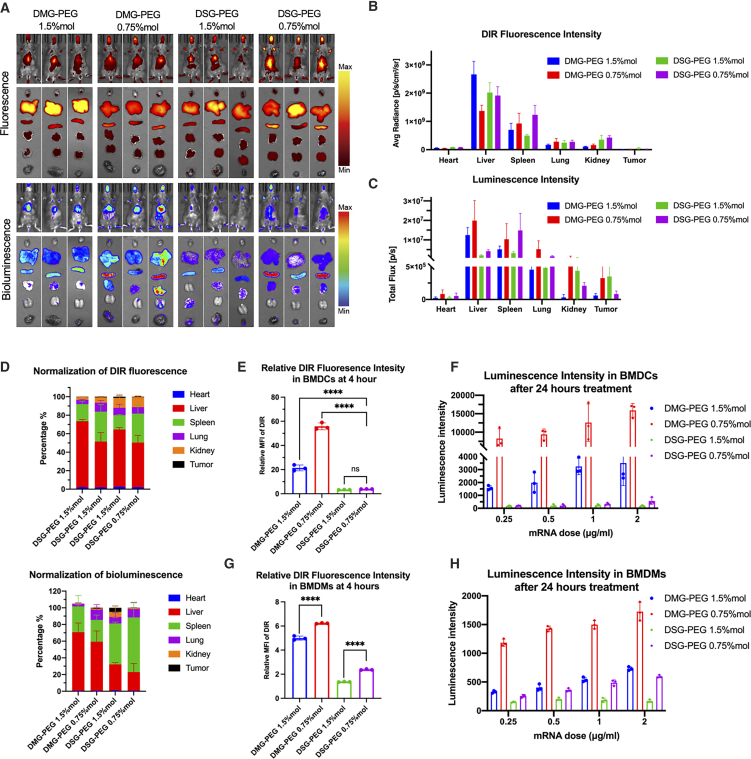
Long-chain PEGylated lipid LNPs at low molar ratio (DSG-PEG 0.75% mol) enhanced spleen accumulation and mRNA delivery *in vivo* (A) Representative fluorescence and bioluminescence imaging (*in vivo* and *ex vivo*) depicting biodistribution and luciferase expression across major organs and tumors from melanoma-bearing mice. (B) Quantification of DiR fluorescence intensity in isolated organs. (C) Luminescence intensity measurements (indicating luciferase activity) in isolated organs. (D) Normalized distribution (%) of DiR fluorescence signals (top) and bioluminescence signals (bottom) across organs. (E–H) Assessment of cellular uptake and transgene expression in immune cells: I DiR fluorescence intensity within BMDCs after 4-h incubation; (F) Luciferase expression intensity within BMDCs measured 24 h post-incubation across varying mRNA doses; (G) DiR fluorescence intensity in BMDMs after 4-h incubation; (H) Luciferase expression intensity in BMDMs evaluated 24 h post-incubation at multiple mRNA doses.

To evaluate this, B16-OVA melanoma mouse models were established by subcutaneous injection of 7 × 10^6^ B16-OVA cells, allowing tumors to form over a 10-day period. Following successful tumor development, mice were intravenously administered four distinct DiR-labeled LNPs formulations loaded with mRNA (eGFP-mRNA: Luc-mRNA = 1:1), total dose: 0.5 mg/kg. Major organs (heart, liver, spleen, lung, and kidney) and tumor tissues were harvested 24 h post-injection for analysis. DiR fluorescence was employed to monitor LNPs distribution, whereas bioluminescence measurements assessed luciferase expression levels, thereby indicating mRNA delivery efficacy (Figure 3A). Quantitative analysis of DiR fluorescence revealed predominant accumulation of all four LNPs formulations in liver and spleen tissues. DMG-PEG 1.5% mol LNPs demonstrated notably higher liver accumulation, whereas the DSG-PEG 0.75% mol formulation—characterized by low PEG molar ratio and slower PEG shedding kinetics—displayed significantly enhanced spleen-specific accumulation (Figure 3B).

Further quantification of luciferase luminescence intensity across isolated organs confirmed robust hepatic mRNA delivery by DMG-PEG LNPs, particularly the DMG-PEG 1.5% mol formulation, accounting for over 60% of normalized bioluminescent signals (Figure 3D). Conversely, DSG-PEG-based formulations showed superior splenic targeting, with the DSG-PEG 0.75% mol LNPs achieving approximately 65% of normalized spleen-specific luminescence signals (Figure 3D).

Interestingly, DMG-PEG 0.75% mol LNPs displayed a more broadly distributed pattern of mRNA expression, with considerable signals detected not only in the liver and spleen but also in the lung tissue. This distribution likely resulted from faster shedding of PEG chains, facilitating rapid formation of a protein corona and subsequent broader organ uptake. Meanwhile, DSG-PEG 1.5% mol LNPs exhibited the weakest mRNA expression in all examined tissues, probably due to the dense PEG surface coverage impeding efficient cellular uptake. Collectively, these *in vivo* biodistribution studies indicate that long-chain PEGylated lipid LNPs at low molar ratio (DSG-PEG 0.75% mol) significantly enhance spleen-specific accumulation and mRNA delivery, suggesting their strong potential for targeted RNA therapeutic applications.

Given these observations, we further investigated whether splenic immune cells preferentially internalized DSG-PEG 0.75% mol LNPs. Since macrophages and DCs represent primary cell types mediating exogenous particle uptake, bone marrow cells from femurs and tibias of 6–8-week-old C57BL/6 mice were collected and differentiated *in vitro* into BMDCs and BMDMs using specific cytokine supplementation.

After differentiation for 7–10 days, cells were re-plated and subsequently exposed to different LNPs formulations for uptake assessment and evaluation of mRNA delivery efficiency. Fluorescence quantification indicated that DMG-PEG 0.75% mol LNPs demonstrated notably higher nanoparticle uptake and luciferase expression within BMDCs, likely owing to rapid PEG shedding enabling greater cellular interactions. Conversely, both DSG-PEG formulations exhibited consistently low uptake and minimal mRNA expression in BMDCs, irrespective of PEG molar ratios, indicating limited cellular internalization under these *in vitro* conditions (Figures 3E and 3F).

BMDMs analyses exhibited analogous trends to those observed in BMDCs, though overall uptake and mRNA expression levels were generally lower across all formulations. This finding suggests DCs might possess a greater inherent capacity for nanoparticle internalization and processing compared to macrophages under the evaluated *in vitro* conditions (Figures 3G and 3H).

Importantly, these *in vitro* results contrasted markedly with our *in vivo* biodistribution findings, where DSG-PEG 0.75% mol LNPs preferentially targeted the spleen. This inconsistency emphasizes a limited direct correlation between *in vitro* cellular uptake assays and actual *in vivo* biodistribution patterns, a disparity also documented in prior LNPs studies. These discrepancies may arise from the lack of physiological complexity in i*n vitro* systems, including the absence of protein corona formation, organ-specific clearance mechanisms, and immune microenvironment interactions that critically influence nanoparticle behavior *in vivo*.^30^^,^^31^

### Long-chain PEGylated lipid LNPs at low molar ratio (DSG-PEG 0.75% mol) facilitated splenic DC uptake and mRNA delivery

Building on prior findings that DSG-PEG 0.75% mol LNPs exhibits spleen-selective accumulation and expression, we further investigated the immune cell-level uptake and gene expression profiles of the four LNPs formulations *in vivo*. Specifically, we sought to evaluate whether the DSG-PEG 0.75% mol formulation enabled efficient mRNA delivery into antigen-presenting cells in the spleen, which is critical for initiating downstream immune responses.

To this end, B16-OVA melanoma-bearing mice were established by subcutaneously inoculating 7 × 10^6^ B16-OVA cells. After tumor development for 10 days, mice received intravenous administration of DiR-labeled LNPs encapsulating enhanced green fluorescent protein (eGFP) mRNA at a dosage of 0.25 mg/kg. Spleens were harvested 24 h post-injection, followed by mechanical dissociation and antibody staining for flow cytometric analysis. DCs and macrophages were identified using specific surface markers: CD11c and MHC-II for DCs, and F4/80 and CD11b for macrophages. LNPs uptake and mRNA translation efficiency were evaluated based on DiR fluorescence and eGFP expression, respectively.

**Figure 4:**
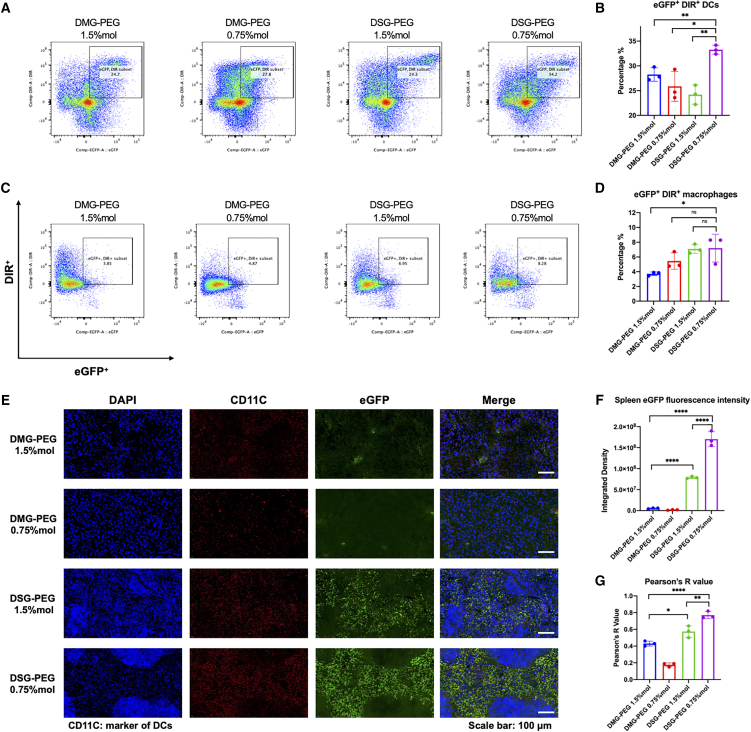
Spleen-targeted DSG-PEG 0.75% mol LNPs enhanced uptake and transgene expression in splenic dendritic cells and macrophages (A) Flow cytometry analysis of splenic DCs harvested 24 h after intravenous administration of DiR-labeled eGFP-mRNA LNPs, showing the proportion of cells positive for both eGFP expression and DiR uptake (eGFP^+^DiR^+^). (B) Quantification of eGFP^+^DiR^+^ DC populations shown in (A). (C) Flow cytometry analysis of splenic macrophages collected at 24 h post-LNPs injection, evaluating the percentage of eGFP^+^DiR^+^ macrophages. (D) Quantification of eGFP^+^DiR^+^ macrophages shown in (C). Detailed gating strategies are provided in the Figure S3. (E) Representative immunofluorescence microscopy images of spleen sections 24 h after LNPs injection, stained for DAPI (blue, nuclei), CD11c (red, DC marker), and eGFP (green, mRNA expression). Scale bar, 100 μm. (F) Quantification of eGFP fluorescence intensity in spleen sections from three randomly selected fields per group by ImageJ. (G) Pearson’s correlation coefficient analysis between CD11c and eGFP signals from three randomly selected fields per group, indicating colocalization of mRNA expression within DCs. Statistical significance was determined using one-way ANOVA with Tukey’s multiple. Comparisons test; ∗*p* < 0.05, ∗∗*p* < 0.01, ∗∗∗∗*p* < 0.0001.

Flow cytometry results revealed distinct populations of DiR^+^eGFP^+^ DCs in all four LNPs treatment groups (Figure 4A). Among them, DSG-PEG 0.75% mol LNPs induced the highest proportion of double-positive DCs, averaging ∼32.5% of the total DC population (Figure 4B). This proportion was significantly higher compared to the other three formulations, indicating superior LNPs uptake and mRNA expression in splenic DCs. For macrophages, the proportion of DiR^+^eGFP^+^ cells remained consistently lower than in DCs across all groups (Figures 4C and 4D), with values below 10%, while DCs showed over 20% positivity in all formulations. Notably, DSG-PEG 0.75% mol LNPs resulted in a significantly higher DiR^+^eGFP^+^ macrophage population compared to DMG-PEG 1.5%, but differences with DMG-PEG 0.75% mol and DSG-PEG 1.5% mol were not statistically significant. These results suggest that DCs are the predominant splenic cell population responsible for nanoparticle uptake and mRNA translation following LNPs administration.

To further corroborate these findings at the tissue level, we performed immunofluorescence staining on spleen sections collected 24 h post-injection. CD11c staining was used to mark DCs, while eGFP expression served as a readout for successful mRNA delivery and translation (Figure 4E). Consistent with the flow cytometry results, the DSG-PEG 0.75% mol group exhibited the strongest eGFP signal within spleen tissue, as shown in both representative images and quantification of integrated fluorescence intensity (Figure 4F). This signal was significantly higher than that observed in any of the other three treatment groups.

Moreover, to evaluate the spatial correlation between DC localization and mRNA expression, we calculated the Pearson’s correlation coefficient (R value) between CD11c (red channel) and eGFP (green channel) signals. DSG-PEG 0.75% mol LNPs demonstrated the highest R value among all formulations, indicating the strongest colocalization between DCs and transgene expression (Figure 4G). This result confirms the preferential delivery of mRNA into splenic DCs by the DSG-PEG 0.75% mol formulation.

In addition, IF of spleen sections revealed a higher abundance of CD11c^+^ cells in the DSG-PEG 0.75% mol group (Figure 4E), suggesting efficient splenic recruitment or retention of DCs following LNPs administration, although further quantification would be needed to confirm this observation.

### Long-chain PEGylated lipid LNPs at low molar ratio (DSG-PEG 0.75% mol) induced adaptive immune responses and suppressed melanoma progression.

Building upon our previous findings that DSG-PEG 0.75% mol LNPs preferentially accumulate in the spleen and are efficiently taken up by DCs, we further evaluated whether this formulation could serve as a vehicle for tumor antigen mRNA delivery and elicit functional adaptive immune responses *in vivo*. Ovalbumin (OVA), a well-established model antigen, was used to construct OVA-mRNA-loaded LNPs and assess their antitumor efficacy and immunogenicity.

**Figure 5:**
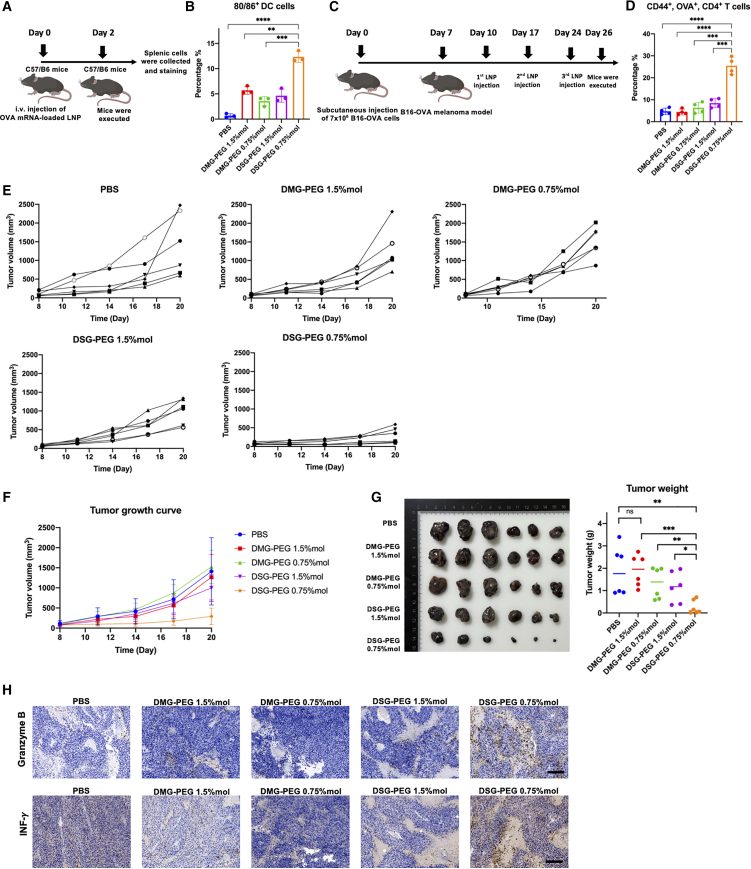
DSG-PEG 0.75% mol OVA-mRNA LNPs activate DCs and suppress melanoma progression (A) Schematic illustration of experimental design for analyzing DCs activation. C57BL/6 mice were intravenously injected with OVA-mRNA-loaded LNPs, and spleen cells were analyzed 24 h later. (B) Flow cytometry quantification of activated DCs (CD80^+^CD86^+^) from spleens corresponding to the experimental design in (A). Detailed gating strategies are provided in the Figure S4. (C) Timeline illustrating the melanoma tumor vaccination study. Mice were subcutaneously inoculated with 7 × 10ˆ5 B16-OVA cells on day 0, followed by vaccinations on days 10, 17, and 24 using different PEGylated OVA-mRNA LNPs formulations. (D) Flow cytometry analysis of antigen-specific memory T cells (CD44^+^OVA^+^CD8^+^) in spleens harvested on day 27. Detailed gating strategies are provided in the Figure S5. (E) Individual tumor growth curves (spaghetti plots) from day 8 to day 20, with tumor volume measured every three days. (F) Tumor growth kinetics summarized as average tumor volumes across treatment groups. (G) Representative tumor photographs and quantitative analysis of tumor weights at endpoint (day 27). (H) IHC staining of tumor tissues for granzyme B and IFN-γ expression at day 27 (representative images). Scale bar: 200 μm. Statistical analysis was performed by one-way ANOVA with Tukey’s multiple comparisons test; ∗*p* < 0.05, ∗∗*p* < 0.01, ∗∗∗*p* < 0.001, ∗∗∗∗*p* < 0.0001, ns = not significant.

Splenic DC activation was evaluated by quantifying the proportion of CD80^+^CD86^+^ double-positive cells within the CD11c^+^MHC-II^+^ population following single intravenous injection of OVA-mRNA-loaded LNPs in C57BL/6 mice (Figure 5A). As shown in Figure 5B, DSG-PEG 0.75% mol LNPs induced the most robust DC activation, with a significantly higher percentage of activated DCs compared to all other groups. This indicates that DSG-PEG 0.75% mol LNPs not only achieve efficient splenic delivery but also effectively promote functional maturation of antigen-presenting cells, a critical prerequisite for initiating downstream adaptive immunity.

To evaluate antigen-specific T cell responses and antitumor effects in a therapeutic context, a B16-OVA melanoma model was established (Figure 5C). Female C57BL/6 mice were subcutaneously inoculated with 7 × 10^5^ B16-OVA cells on day 0. Mice were randomly assigned into four treatment groups (*n* = 6 per group) and received three intravenous injections of OVA-mRNA-loaded LNPs on days 10, 17, and 24, respectively. On day 26, spleens were harvested and processed into single-cell suspensions. Flow cytometry was used to assess the percentage of antigen-specific memory CD4^+^ T cells (CD44^+^OVA^+^CD4^+^) within the CD45^+^CD3^+^ T cell population. As shown in Figure 5D, DSG-PEG 0.75% mol LNPs significantly increased the proportion of CD4^+^CD44^+^OVA^+^ T cells compared to the other three formulations, confirming that this LNPs formulation effectively promotes antigen-specific T cell priming following repeated mRNA vaccination.

During the course of treatment, tumor growth and body weight were monitored by digital caliper every 2–3 days. Tumor growth data from individual mice (Figure 5E) and average tumor volume curves (Figure 5F) showed that only the DSG-PEG 0.75% mol group exhibited significant tumor growth inhibition. In contrast, mice treated with either DMG-PEG 1.5% mol or DMG-PEG 0.75% mol LNPs showed no observable difference in tumor progression compared to the PBS control group, indicating limited therapeutic efficacy from these formulations.

Terminal tumor burden was further evaluated by visual inspection and weighing of excised tumors on day 27 (Figure 5G). Consistent with tumor growth curves, the DSG-PEG 0.75% mol group showed a marked reduction in tumor size and mass. Quantitative analysis of tumor weights demonstrated statistically significant differences between the DSG-PEG 0.75% mol group and all other groups, with three out of six mice in this group showing clear tumor regression.

To further assess the local immune activation within the tumor microenvironment, we performed immunohistochemical staining for granzyme B and interferon-gamma (IFN-γ) (Figure 5H). Compared to PBS and DMG-PEG formulations, the DSG-PEG 0.75% mol group exhibited markedly higher levels of both markers. Granzyme B staining revealed intense and widespread cytotoxic lymphocyte infiltration, indicative of enhanced cytolytic activity. Similarly, IFN-γ expression was substantially elevated in the DSG-PEG 0.75% mol group, suggesting robust Th1-type immune responses and increased proinflammatory signaling. These results support the notion that spleen-targeted LNPs with optimized PEG composition (DSG-PEG 0.75% mol) more effectively stimulate cytotoxic T cell activity and immune-mediated tumor rejection. Consistent with the qualitative staining results in Figure 5H, semi-quantitative analysis of immunohistochemistry (IHC) staining showed that the DSG-PEG 0.75% mol group had significantly higher positive areas for both granzyme B and IFN-γ compared to other groups (Figure S6).

To assess the *in vivo* safety of these LNP formulations, organ weights and histopathological analyses were conducted after three repeated intravenous injections. No significant changes in body weight were observed across all treatment groups during the study period, indicating that repeated intravenous administration of OVA-mRNA LNPs with different PEGylated lipid compositions was well tolerated in mice (Figure S7). As shown in Figure S8, there were no significant changes in the weights of major organs (heart, liver, spleen, lung, and kidney) across all treatment groups compared to PBS controls. Furthermore, H&E staining of liver and spleen tissues (Figure S9) revealed no observable tissue damage, necrosis, or inflammatory infiltration, indicating that repeated administration of these PEGylated lipid modified LNPs did not induce detectable organ toxicity.

In summary, DSG-PEG 0.75% mol LNPs demonstrated superior performance in delivering tumor antigen mRNA, activating immune responses, and inhibiting tumor progression. These findings highlight the potential of LNPs formulated with long-chain PEGylated lipids at low molar ratio as a promising platform for mRNA-based cancer immunotherapy.

## Discussion

This study demonstrates that rational modulation of PEGylated lipid composition—specifically, the incorporation of long-chain DSG-PEG at a low molar ratio (0.75%)—effectively redirects LNP biodistribution from the liver to the spleen, thereby enhancing splenic mRNA delivery and promoting potent antitumor immunity.

The observed spleen tropism likely results from the combined influence of nanoparticle physicochemical attributes and protein interactions. Reduced PEG density increased zeta potential and particle size, while slower PEG shedding altered protein-corona profiles. Proteomic analysis revealed markedly reduced ApoE adsorption on DSG-PEG LNPs compared with conventional DMG-PEG formulations. Given ApoE’s role in LDLR-mediated hepatic uptake, its diminished association likely contributes to the attenuated liver accumulation and enhanced splenic targeting observed here.

Factors such as protein-corona composition, total adsorbed protein, and pharmacokinetic behavior collectively determine LNP fate; however, their causal relationships remain difficult to disentangle. These processes form a complex “black box” in nanoparticle biology. Our study links several of these correlated events—PEG composition, corona remodeling, and biodistribution shift—to provide an integrative view of how PEG modulation governs spleen targeting. Among the identified corona proteins, albumin may prolong circulation and facilitate immune-organ exposure through passive transport, whereas ApoA1 and ApoA4, as HDL components, may influence lipid exchange and receptor-mediated uptake, contributing to tissue distribution. We also acknowledge that differences between *in vitro* and *in vivo* corona formation may limit direct correlation of results; such discrepancies likely arise from variations in plasma composition and flow dynamics. Moreover, the protein-corona profiling was performed once, aiming to qualitatively compare compositional differences rather than quantify specific protein contributions, which also represents a limitation of our study. In particular, the use of EDTA plasma *in vitro*, while preventing coagulation-related interference, may suppress complement activation and thereby underestimate complement-mediated interactions with splenic cells *in vivo*. Future studies will further elucidate these context-dependent effects to refine mechanistic understanding.

Interestingly, DSG-PEG 0.75% LNPs displayed limited uptake by bone-marrow-derived DCs (BMDCs) *in vitro* yet achieved robust mRNA expression in splenic DCs *in vivo*. This apparent discrepancy likely reflects the absence of dynamic corona formation and flow-mediated transport in cell cultures. Moreover, the spleen’s open sinusoidal structure and dense phagocyte network facilitate nanoparticle retention and uptake, collectively accounting for the efficient *in vivo* DC targeting observed.

Functionally, DSG-PEG 0.75% mol LNPs effectively activated splenic DCs, increased antigen-specific CD4^+^ T cell responses, and significantly suppressed melanoma growth. Enhanced intratumoral granzyme B and IFN-γ levels confirmed potent adaptive antitumor immunity.

Although repeated dosing of PEGylated nanoparticles can sometimes trigger anti-PEG antibodies and accelerated blood clearance (ABC), our regimen involved only three intravenous administrations, which achieved strong antitumor efficacy. Therefore, the risk of pronounced anti-PEG immune activation under these conditions is expected to be minimal.^32^

Moreover, while both spleen and lymph nodes are critical secondary lymphoid organs involved in immune priming, their targeting requires distinct delivery strategies. Notably, intravenous delivery tends to elicit stronger immune activation than intramuscular routes, suggesting that our spleen-targeting strategy holds promise for enhancing tumor vaccine efficacy.^33^^,^^34^

Collectively, these findings substantiate the feasibility and effectiveness of engineering PEGylated lipid composition as a straightforward yet powerful strategy to modulate LNPs biodistribution. Mechanistically, we propose that the observed spleen-targeted biodistribution of DSG-PEG 0.75% mol LNPs emerges from an interplay of thermodynamic factors (such as particle size and surface charge) and kinetic processes (including PEG shedding dynamics and protein corona formation).

In conclusion, this study presents a straightforward and effective strategy for spleen-targeted mRNA delivery by tuning PEGylated lipid composition. The optimized DSG-PEG 0.75% formulation directs LNPs to the spleen, activates DCs, and elicits strong antigen-specific T cell responses, leading to potent antitumor immunity. This approach requires only simple adjustment of PEG-lipid ratios during standard microfluidic mixing, enabling scalable manufacturing and facilitating clinical translation of spleen-targeted mRNA therapeutics.

## Materials and methods

### Ethics statement

All animal procedures were performed in compliance with institutional guidelines and approved by the Institutional Animal Care and Use Committee (IACUC) of Tsinghua University (approval no: 16-QF1.G23-2).

### Materials

Heptadecan-9-yl 8-((2-hydroxyethyl) (6-oxo-6-(undecyloxy)hexyl)amino) octanoate (SM-102), 1,2-distearoyl-sn-glycero-3-phosphocholine (DSPC), cholesterol (Chol), and 1,2-dimyristoyl-rac-glycero-3-methoxypolyethylene glycol-2000 (DMG-PEG2000) were purchased from AVT Pharmaceutical Tech Co., Ltd. Distearoyl-rac-glycero-PEG2000 (DSG-PEG2000) was custom-synthesized by JenKem Technology. Firefly luciferase mRNA (Luc-mRNA) and enhanced green fluorescent protein mRNA (eGFP-mRNA), both ARCA capped and chemically modified with 3′-OMe-m7G and N1-methyl-pseudouridine, were obtained from Yeasen Biotechnology. 1,1-dioctadecyl-3,3,3,3-tetramethylindotricarbocyanine iodide (DiR), citrate buffer, Tris buffer (Ruixinnuo Biology), and nuclease-free water (Thermo Fisher) were used as received.

Antibodies used in this study were listed in Tables S4–S6. Chemical structures of lipids used in this study were listed in Table S7.

### mRNA-loaded LNPs preparation and characterization

mRNA-loaded LNPs were formulated via a microfluidic-assisted nanoprecipitation method as previously described.^35^ SM-102, Chol, DSPC, and PEGylated lipids (DMG-PEG2000 or DSG-PEG2000) were dissolved in ethanol at molar ratios of 50:38.5:10:1.5 or 50:39.25:10:0.75. This lipid solution was rapidly mixed with 10 mM sodium acetate buffer (pH 4.0) containing Luc or eGFP mRNA using a microfluidic device (LNP-S1, FluidicLab). The N/P ratio (nitrogen from ionizable lipid to phosphate from mRNA) was set at 6:1.

Ethanol was removed via ultracentrifugal (100 kDa MWCO, Millipore) at 2,000 × g, 4°C, followed by buffer exchange with 5 mM Tris buffer and 300 mM sucrose at target pH 7.4. Final LNPs were sterilized by filtration through a 0.2 μm PES membrane and stored at −80°C. DiR-labeled LNPs were prepared by incorporating 0.5 mol % free DiR dye into the ethanol phase.

LNPs size was measured by DLS using a Zetasizer Nano ZS90 (Malvern). Encapsulation efficiency (EE %) was assessed using the Quant-iT RiboGreen assay (Thermo Fisher). Total mRNA content was determined by lysing LNPs with 1% Triton X-100, while untreated samples reflected free, unencapsulated mRNA. Fluorescence intensity (Ex/Em: 480/520 nm) was measured, and EE % was calculated as follows:

(Equation 1) $$EE\;\%=\frac{\left(F_{t}-F_{u}\right)}{F_{t}}\times 100$$

where F_t_ and F_u_ represent total and unencapsulated mRNA fluorescence, respectively. Microstructure was characterized by cryo-EM (200 kV, FEI).

### LNPs protein corona isolation

Mouse plasma collected with EDTA as anticoagulants was used to form the protein corona. To isolate protein corona, DiR-labeled LNPs were incubated with mouse plasma at a 1:1 ratio (v/v) at 37°C for 4 h. The mixtures were layered beneath a three-step sucrose gradient (30%, 15%, and PBS) and ultracentrifuged at 200,000 × g for 3 h at 4°C (Optima MAX-XP, Beckman Coulter). Control samples with plasma only or LNPs only were included.

A schematic diagram of the sucrose density gradient ultracentrifugation procedure is shown in Figure S10 to illustrate the separation principle, in which LNPs associated with protein corona are enriched in the top fraction (Fraction 1) and free serum proteins are retained in lower fractions. These fractions were used for downstream SDS-PAGE and proteomic analysis.

### SDS-PAGE for LNPs corona

50 μL of mRNA-loaded LNPs protein corona sample were loaded into the wells of 4%–12% precast protein gel (Yeasen). Mouse plasma and mouse albumin was loaded as a control. The gel was run starting at 150 V, 2 h. Afterward, the gel was disassembled and immersed in commassie blue fast stain solution (Yeasen) for 10 min, after the gels washed in MilliQ water under gently shaking at room temperature, and imaged with gel imaging systems (Bio-Rad).

### Reduction, alkylation, and digestion of proteins

Protein lysates were processed by the FASP protocol. Briefly, 100 μg of sample was reduced with 10 μL of 1M dithioerythritol (DTT) at 35°C, 800 rpm shaking, 30 min, then then alkylated with 10 μL of 1 M iodoacetamide (IAM) in the dark for 30 min at room temperature, 800 rpm shaking. Then mixed with 200 μL of 8 M urea in 0.1 M Tris/HCl, pH 8.5 (UA), in the Microcon-10 kDa centrifugal ultrafiltration and then centrifuged at 14000 g, 10 min, washed with 200 μL 50 mM NH4HCO3. The eluates were discarded, then 200 μL 2 M UA buffer, 50 μL 50 mM NH_4_HCO_3_, and 2 μL 100 mM CaCl_2_ pipetted into the filtration unit. 1 μg/mL tyrosine was added to the filters, enzyme to protein ratio of 1:100, and samples were incubated in darkness overnight. Next day, 50 μL 0.1% formic acid (FA) was added to terminate reaction, the released peptides were collected by centrifugation at 14 000 g for 10 min followed by two washes with 50 μL of deionized water. Combined eluents and dried, and dissolved in 0.1% FA at an average sample concentration of 500 ng/μL.

### LC-MS for LNPs corona

The protein corona samples were analyzed by ultra-performance liquid chromatography coupled with tandem mass spectrometry (UPLC–MS/MS) using a Vanquish *Neo* nanoLC system (Thermo Fisher Scientific) interfaced with a Q Exactive Plus (QE Plus) mass spectrometer. Peptide separation was performed on a 75 μm × 25 cm analytical C18 column (Vanquish *Neo* nanoLC column) with a 75 μm × 2 cm Acclaim PepMap 100 trap column. Samples (2 μL, ∼1000 ng peptides) were loaded and eluted using a 100-min linear gradient from 4% to 99% mobile phase B (90% acetonitrile with 0.1% formic acid) at a flow rate of 300 nL/min. Mobile phase A consisted of water with 0.1% formic acid. The nanoLC gradient conditions are summarized in Table S1. Raw MS data were processed using Proteome Discoverer 2.5 (Thermo Fisher Scientific). Database searching was performed against a decoy version of the UniProt mouse proteome database. Trypsin was specified as the digestion enzyme. Carbamidomethylation of cysteine residues (+57.021 Da) was set as a fixed modification, and methionine oxidation (+15.995 Da) was included as a variable modification.

### *In vitro* differentiation of bone marrow-derived dendritic cells and bone marrow-derived macrophages and mRNA-LNPs treatment

Bone marrow cells were harvested from the femurs and tibias of 6–8-week-old C57BL/6 mice under sterile conditions. For BMDCs differentiation, cells were cultured in RPMI 1640 medium supplemented with 10% FBS, 1% glutamine, 50 mM HEPES, 10 ng/mL GM-CSF, and 10 ng/mL IL-4. For BMDMs induction, M-CSF (10 ng/mL) was used instead of GM-CSF and IL-4. Cells were plated at 1 × 10^6^ cells/mL in 6-well plates and maintained at 37°C in a 5% CO_2_ incubator. Half of the culture medium was replaced with fresh cytokine-supplemented medium every 2–3 days. Immature BMDCs were collected on day 6–7 by gentle pipetting, while adherent BMDMs were harvested by trypsinization on day 8–10.

Differentiated BMDCs and BMDMs were treated *in vitro* with DiR-labeled LNPs containing Luc-mRNA. During *in vitro* transfection, cells were cultured in complete medium supplemented with 10% FBS, consistent with standard cell-culture practice. Four hours post-treatment, DiR fluorescence intensity was analyzed by flow cytometry to assess nanoparticle uptake. Luciferase expression was quantified 24 h after treatment, lysed with Glo lysis buffer, and incubated with luciferin potassium salt (150 ng/mL). Bioluminescence was measured using a microplate luminometer.

### LNPs distribution and expression

The Dir labeled mLuc-loaded LNPs were injected into the KPC bearing C57 BL/6 mouse model through i.v. injection at an mRNA dosage of 0.5 mg/kg. 24 h, D-Luciferin (150 mg/kg) was injected intraperitoneally, and the mice were rapid aesthesia via 5% isoflurane. Fluorescence (Ex/Em 740/780) and bioluminescence imaging using an IVIS spectrum imaging system (Caliper Life Sciences). Imaging was repeated at 24 h, animals euthanized, and organs collected for *ex vivo* imaging. Then animals euthanized, main organs heart, liver, spleen, lung, kidney, and tumor were collected for *ex vivo* imaging. Image acquisition for live animals and excised organs was initiated at 10 min and 15 min post-D-luciferin administration, respectively. Exposure times for whole animal and organs were 30 s. Quantification of fluorescence and bioluminescence was achieved by calculating photon flux within the region of interest (ROI) using the Living IMAGE software package (Caliper).

### *In vivo* spleen uptake and expression analysis of DiR-labeled mRNA-LNPs in DCs and macrophages

C57BL/6 mice were intravenously injected with DiR-labeled lipid nanoparticles encapsulating a 1:1 molar ratio of eGFP-mRNA and Luc-mRNA at a total dose of 0.5 mg/kg. After 24 h, spleens were collected and processed into single-cell suspensions. Briefly, spleens were passed through 70 μm cell strainers in cold PBS with 1% FBS, followed by red blood cell lysis using ammonium chloride lysis buffer. The resulting cells were washed, filtered through sterile gauze, and resuspended in PBS at a concentration of 1–2 × 10^7^ cells/mL.

Cells were stained for viability (Zombie NIR) and blocked with anti-CD16/32 Fc receptor antibody. For DC and macrophage identification, the following fluorophore-conjugated antibodies were used: CD11c-PE, MHC-II–PE/Cy7 (for DCs), and F4/80–BV421, CD11b–APC (for macrophages). DiR signal (740/780 nm) and eGFP fluorescence (488/520 nm) were measured to assess LNPs uptake and mRNA expression, respectively. Flow cytometry gating was performed sequentially on live single cells, followed by identification of myeloid cells (CD3^-^ B220^-^), and further delineation of CD11c^+^ MHCII^+^ DCs and F4/80^+^ macrophages. The proportion of double-positive cells (DiR^+^eGFP^+^) within each subset was quantified to evaluate both LNPs uptake and mRNA transgene expression. Flow cytometry gating strategy is provided in the Figure S3.

### *In vivo* assessment of DCs activation in the spleen following mRNA-LNPs treatment

To evaluate DCs activation *in vivo*, C57BL/6 mice were intravenously injected with OVA mRNA-loaded LNPs at a total mRNA dose of 0.25 mg/kg. After 48 h, mice were euthanized, and spleens were dissected and collected. Spleens were mechanically dissociated and passed through 70 μm cell strainers using cold PBS containing 1% FBS. Red blood cells were lysed with ammonium chloride buffer, followed by centrifugation, washing, and filtration through sterile gauze. Cells were resuspended in PBS and adjusted to a final concentration of 1–2 × 10^6^ cells/mL.

For flow cytometry analysis, cells were pre-blocked with anti-CD16/32 (Fc block) and stained with a panel of antibodies including CD45 (eFluor 450), CD11c (APC), MHCII (BV421), CD80 (PE-Cy7), and CD86 (FITC). Samples were fixed prior to acquisition. The percentage of activated DCs (defined as CD11c^+^MHCII^+^CD80^+^CD86^+^ cells within the CD45^+^ live population) was analyzed by flow cytometry. Flow cytometry gating strategy is provided in the Figure S4.

### Flow cytometric analysis of OVA^+^ CD4^+^ T cells in the spleen after LNPs treatment

To assess antigen-specific T cell responses, C57BL/6 mice were subcutaneously inoculated with 7 × 10^6^ B16-OVA melanoma cells on day 0. Mice were treated with OVA mRNA-loaded LNPs on days 10, 17, and 24. On day 26, mice were euthanized and spleens were aseptically collected. Spleens were processed into single-cell suspensions by mechanical dissociation through 70 μm strainers in cold PBS with 1% FBS. Red blood cells were lysed using ammonium chloride lysis buffer, followed by washing and filtration. Cells were resuspended in PBS and adjusted to 1 × 10^7^ cells/mL.

Spleen cells were stained with viability dye (Zombie NIR) and incubated with Fc block (anti-CD16/CD32), followed by staining with the following antibodies: CD45 (PerCP-Cy5.5), CD3 (BV421), CD4 (APC), CD8 (FITC), CD44 (PE), and SIINFEKL OVA-peptide-MHC class II tetramer (eFluor 710). After fixation, samples were analyzed by flow cytometry. The frequency of activated, antigen-specific CD4^+^ T cells was determined by gating on CD45^+^CD3^+^CD4^+^CD44^+^OVA^+^ cells. Flow cytometry gating strategy is detailed in the Figure S5.

### *In vivo* therapeutic evaluation of mRNA-LNPs in the B16-OVA melanoma model

To evaluate the antitumor activity of mRNA-loaded LNPs, a subcutaneous B16-OVA melanoma model was established in female C57BL/6 mice. On day 0, mice were inoculated subcutaneously in the right flank with 7 × 10^6^ B16-OVA cells suspended in 100 μL of sterile PBS. Tumor growth was monitored regularly, and once tumors became palpable (approximately day 7), mice were randomly assigned into treatment groups (*n* = 6 per group). Mice received intravenous injections of OVA mRNA-loaded LNPs at a dose of 0.25 mg/kg total mRNA per injection via the tail vein on days 10, 17, and 24. Control animals received PBS. Tumor size was measured using digital calipers every 2–3 days starting from day 8. Tumor volume was calculated using the standard formula:

(Equation 2) $$V\text{olume}=\left(\text{length}\times {\text{width}}^{2}\right)\;/2$$

where length is the longest diameter and width is the perpendicular shorter diameter. Measurements were recorded until day 26. On day 26, mice were euthanized, and tumors were surgically removed, photographed, and weighed to assess overall tumor burden.

### Statistical analysis

Data are presented as means ± SD. Statistical significance was assessed by one-way ANOVA with Tukey’s multiple comparisons test. A *p* value <0.05 was considered significant. All experiments were performed in at least three biological replicates unless otherwise stated.

## Data and code availability

The data generated in this study are available upon request from the corresponding author.

## Acknowledgments

We thank F. Yang and X. Li for technical support during EM data collection. We thank the Tsinghua University Branch of China National Center for Protein Sciences (Beijing) for providing the cryo-EM facility support and the computational facility support. We also thank H. Li from the Institute of Immunology at Tsinghua University for helpful assistance with experimental details.

## Author contributions

S.L. and L.Z. contributed equally to this work. S.L. conceived the project, performed the major experiments, and wrote the manuscript. L.Z. contributed to the project conception and experimental design. W.W. was responsible for figure preparation and conducting cell experiments. M.C. assisted with animal experiments. F.Q. supervised this research and manuscript preparation, also secured research funds for this study. All authors discussed the results and approved the final manuscript.

## Declaration of interests

L.Z. is currently at the Institute for Drug Delivery & Translational Research, Fuda Biomedical Innovation Institute of Quzhou, Quzhou 324000, China.

## Supplemental information

Document S1. Figures S1–S10 and Tables S1–S7

Document S2. Article plus supplemental information
