# Supplementary material for: Spleen-targeted mRNA delivery via long-chain PEGylated lipids at low molar ratio enhances antitumor immunity against melanoma
Source: Mol Ther Nucleic Acids. 2025 Dec 12;37(1):102803. doi: 10.1016/j.omtn.2025.102803 (PMC12799935; doi:10.1016/j.omtn.2025.102803)
Supplement: Document S1. Figures S1–S10 and Tables S1–S7 [file mmc1.pdf]

OMTN, Volume *37*

Supplemental information

Spleen-targeted mRNA delivery via long-chain PEGylated lipids at low molar ratio enhances antitumor immunity against melanoma

Shiyu Liu, Longlong Zhang, Wenbo Wu, May Yee Chen, and Feng Qian

Supplemental Material

Table S1. Gradient and flow rates for nanoUPLC separation

Time (min)	Phase A（%）	Phase B（%）
0	96	4
1.8	95.5	4.5
2	95	5
54	80	20
78	65	35
81.5	1	99
100	1	99

Table S2. Abundance of top 20 most abundant proteins identified in the protein corona by LC-MS across different LNPs formulations and mouse plasma

	DMG-PEG 1.5 % mol	DMG-PEG 0.75 % mol	DSG-PEG 1.5 % mol	DSG-PEG 0.75 %mol	Mouse Plasma
α-2-HS-Glycoprotein	373,651,885	619,437,157	720,188,597	247,477,830	894,698,981
Albumin	22,582,297,549	23,377,268,970	22,773,663,112	12,287,121,639	46,029,731,676
Apoliprotein A1	32,898,510,480	14,264,767,698	15,320,126,515	31,503,389,229	5,339,274,870
Apoliprotein A2	505,544,287	377,848,822	580,927,060	870,365,688	401,177,915
Apoliprotein A4	15,558,061,205	3,397,240,829	10,824,933,250	32,555,807,464	802,962,289
Apoliprotein B	14,595,794,623	2,126,755,559	3,185,089,237	7,381,328,596	187,249,391
Apoliprotein C1	1,173,593,520	391,951,423	538,997,754	1,522,987,437	50,531,166
Apoliprotein E	9,276,516,370	1,380,780,551	2,035,227,752	6,675,390,263	194,245,111
Complement 3	744,480,753	637,833,867	698,584,462	542,190,803	3,640,382,599
Coagulation factor 2	418,495,684	286,959,608	323,443,297	646,430,044	453,126,683
Hemopexin	827,538,650	1,294,089,606	1,227,138,886	349,015,690	5,350,570,060
Kininogen 1	427,753,457	435,516,033	504,084,238	354,239,718	774,119,428
Keratin 1	796,219,388	152,606,424	99,615,377	464,149,126	54,085,508
Paraoxonase 1	314,345,691	103,709,744	134,465,959	285,854,586	48,145,250
α-2-Macroglobulin	342,015,305	106,411,116	73,437,254	336,415,569	5,077,296,436
Serum amyloid A1	387,352,486	313,043,855	394,815,349	647,499,601	95,912,082
Serum amyloid A2	1,204,137,532	103,136,869	128,320,069	1,406,432,350	11,761,420
α-2-Antitrypsin 1	513,134,945	624,734,017	780,400,659	263,225,363	2,406,527,883
Serpina3k	385,650,743	871,625,575	1,045,580,150	384,809,127	1,781,957,753
Transferrin	1,697,306,210	2,298,598,550	1,621,297,285	778,822,638	17,004,133,688

Table S3. Top 20 most abundant proteins identified in the protein corona by LC-MS across different LNPs formulations and mouse plasma

Protein	DMG1.5	DMG0.75	DSG1.5	DSG0.75	Mouse Plasma
Alpha-2-HS-glycoprotein	0.33	1.06	1.05	0.23	0.73
Albumin	20.01	40.07	33.23	11.59	37.80
Apolipoprotein A-I	29.16	24.45	22.36	29.71	4.38
Apolipoprotein A-II	0.45	0.65	0.85	0.82	0.33
Apolipoprotein A-IV	13.79	5.82	15.80	30.71	0.66
Apolipoprotein B	12.94	3.65	4.65	6.96	0.15
Apolipoprotein C-I	1.04	0.67	0.79	1.44	0.04
Apolipoprotein E	8.22	2.37	2.97	6.30	0.16
Complement C3	0.66	1.09	1.02	0.51	2.99
Prothrombin	0.37	0.49	0.47	0.61	0.37
Hemopexin	0.73	2.22	1.79	0.33	4.39
Kininogen-1	0.38	0.75	0.74	0.33	0.64
Keratin 1	0.71	0.26	0.15	0.44	0.04
Paraoxonase 1	0.28	0.18	0.20	0.27	0.04
Pregnancy zone protein	0.30	0.18	0.11	0.32	4.17
α-2-Macroglobulin<br>Serum amyloid A1	0.34	0.54	0.58	0.61	0.08
Serum amyloid A-2	1.07	0.18	0.19	1.33	0.01
Alpha-1-antitrypsin 1-2	0.45	1.07	1.14	0.25	1.98
Serpina3k	0.34	1.49	1.53	0.36	1.46
Serotransferrin	1.50	3.94	2.37	0.73	13.96

Table S4. Antibodies used for flow cytometry analysis of eGFP⁺DiR⁺ DCs and macrophages

Marker	Dye	Em/Ex	Dilution	Catalog No.	Supplier
CD45	eFlour450	405/445	1:200	48-9459-42	Invitrogen
CD11C	APC	627/640	1:200	550261	Biolegend
MHCII	Brilliant Violet™ 421	406/423	1:200	404-5321-82	Invitrogen
CD80	PE-Cy7	569/780	1:200	104734	Biolegend
CD86	FITC	488/525	1:200	105006	Biolegend
OVA-peptide	eFlour710	482/708	1:200	46-5743-82	Invitrogen

Table S5. Antibodies used for DC activation analysis

Marker	Dye	Em/Ex	Dilution	Catalog No.	Supplier
Zombie NIR	APC/Cyanine7	633/746	1:100-1:1000	423105	Biolegend
CD45	PerCP-Cyanine5.5	489/679	1:200	45-0451-80	Invitrogen
CD3	Brilliant Violet 421™	405/421	1:200	100227	Biolegend
CD4	APC	651/660	1:200	17-0042-82	Invitrogen
CD8	FITC	498/517	1:200	100705	Biolegend
OVA-peptide	eFlour710	482/708	1:200	46-5743-82	Invitrogen
CD44	PE	482/578	1:200	12-0441-82	Invitrogen

Table S6. Antibodies used for antigen-specific $CD4^+$ T cell analysis

Marker	Dye	Em/Ex	Dilution	Catalog No.	Supplier
Zombie NIR	APC/Cyanine7	633/746	1:100-1:1000	423105	Biolegend
CD6/CD32	-	-	1:50	101301	Biolegend
F4/80	Brilliant Violet™ 421	406/423	1:50	404-4801-80	Invitrogen
CD11B	APC	640/660	1:200	101211	Biolegend
CD11C	PE	565/576	1:200	12-0114-81	Invitrogen
MHCII	PE/ Cyanine7	488/532&561	1:200	107611	Biolegend
DIR	DIR	740/780	-	40757ES25	Yeasen
eGFP	eGFP	488/520	-	-	-

Table S7. Chemical structures of lipids used in this study

Lipid name	Chemical structure
SM102	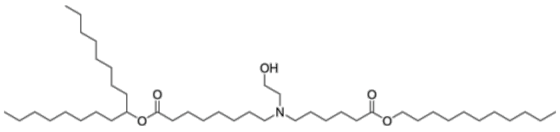
DSPC	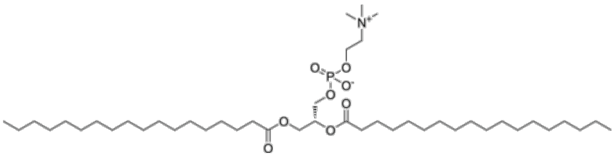
Cholesterol	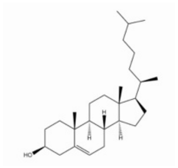
DMG-PEG2k	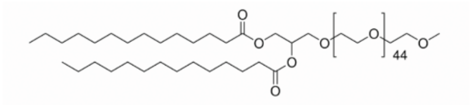
DSG-PEG2k	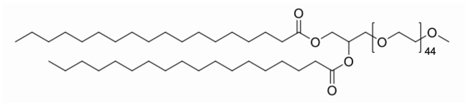

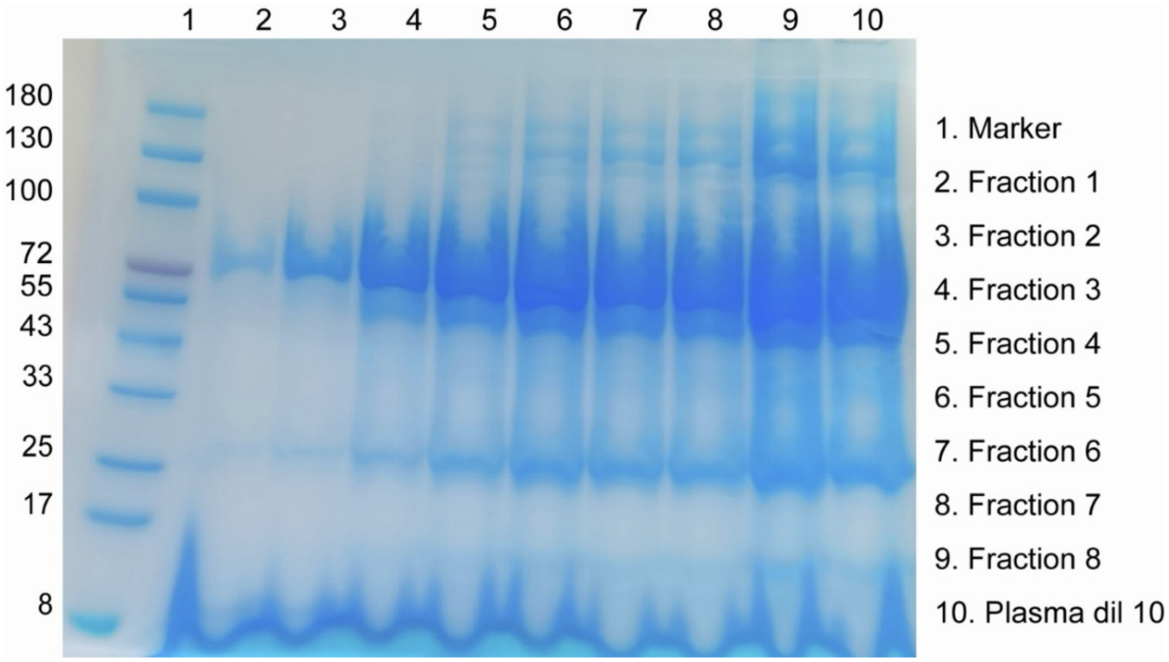

Figure S1. SDS-PAGE analysis of proteins in different fractions after ultracentrifugation of DMG-PEG 1.5 % mol LNPs incubated with mouse plasma.

LNPs were incubated with mouse plasma and subjected to sucrose density gradient ultracentrifugation. The post-centrifugation gradient was divided into eight fractions from top to bottom, followed by SDS-PAGE to analyze the distribution of adsorbed proteins. Lane 1 shows the protein marker, and lanes 2–9 correspond to Fractions 1–8, respectively, while lane 10 contains proteins from untreated plasma as a control. Most proteins were concentrated in Fractions 4–7, whereas Fraction 1 (top layer, enriched with LNPs) showed distinct protein bands, indicating the formation of a protein corona. To avoid interference from free serum proteins in the lower fractions, Fraction 1 was selected for subsequent protein corona characterization and analysis.

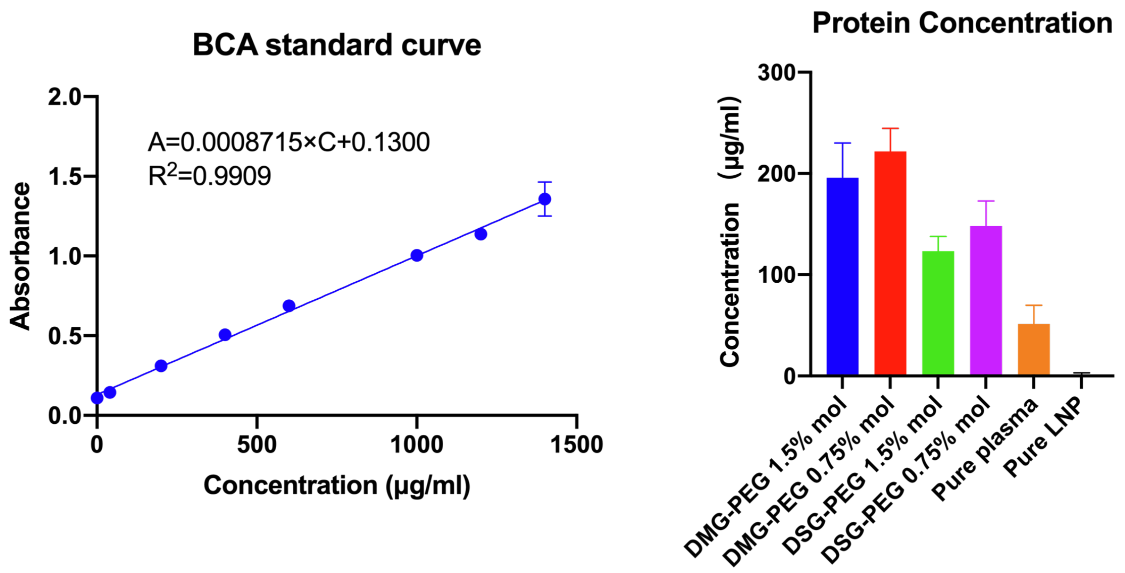

Figure S2. BCA-based quantification of protein corona on LNPs.

Left: BCA standard curve generated using bovine serum albumin (BSA) with a linear regression equation:

$$A = 0.0008715 \times \mathrm{C} + 0.13000\ (\mathrm{R}^2 = 0.9909)$$

where A is absorbance and C is protein concentration (μg/mL).

Right: Total protein content adsorbed on LNPs was measured by BCA assay after plasma incubation and ultracentrifugation. DMG-PEG-formulated LNPs (both 1.5 % and 0.75 %) showed higher protein adsorption than their DSG-PEG counterparts. Pure plasma and pure LNPs (without plasma incubation) served as controls. Data are presented as mean ± SD (n = 3).

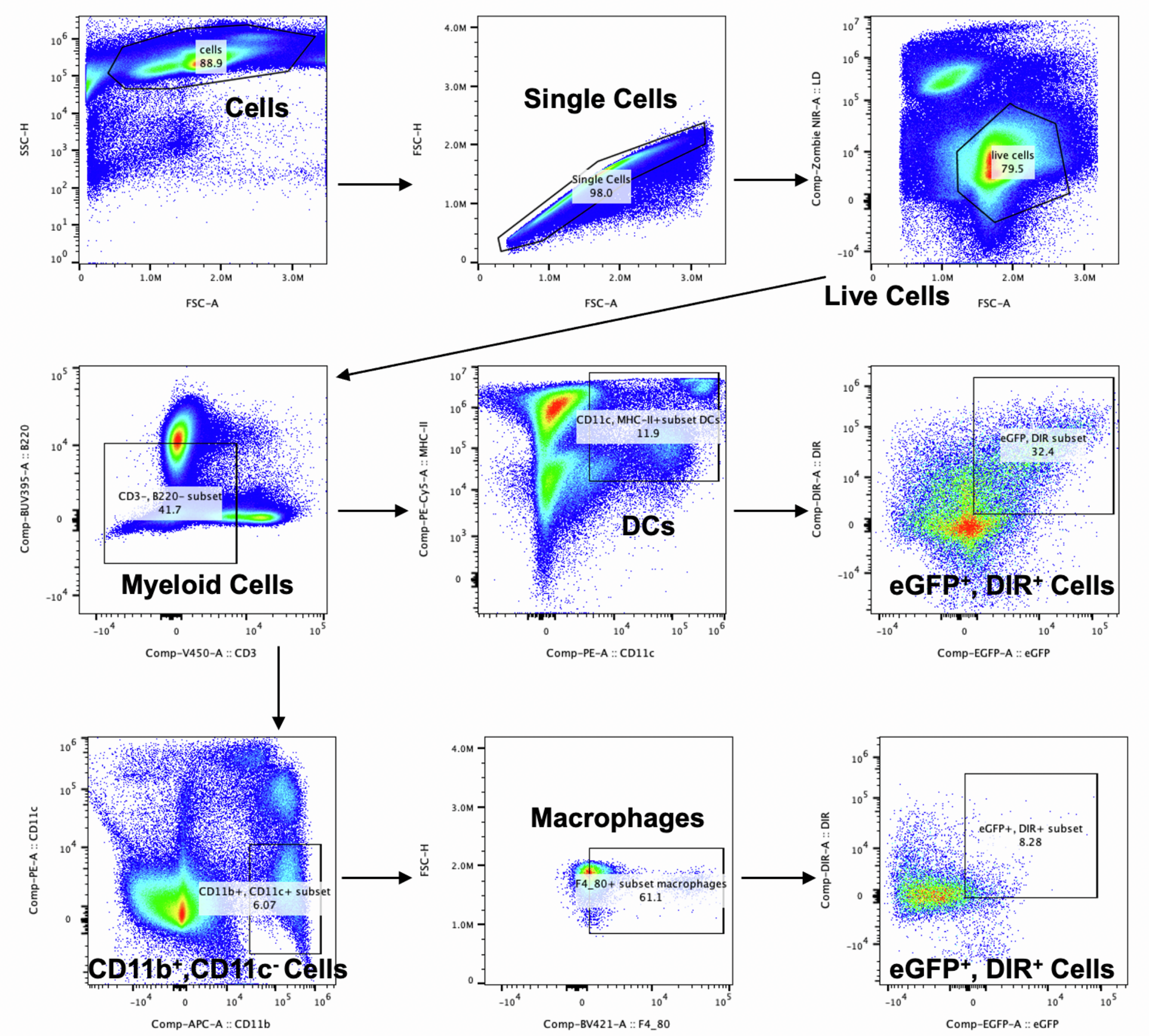

Figure S3. **Flow cytometry gating strategy for identifying eGFP⁺DiR⁺ DCs and macrophages in mouse spleens following intravenous injection of DiR-labeled eGFP-mRNA-loaded LNPs.**

Splenocytes were first gated to exclude debris and doublets, and live cells were identified as Zombie NIR$^-$. Myeloid cells were defined by excluding CD3$^+$ and B220$^+$ populations. DCs were identified as CD11c$^+$MHC-II$^+$, and macrophages were defined as CD11b$^+$F4/80$^+$. Within each subset, eGFP$^+$DiR$^+$ double-positive cells were quantified to indicate successful LNPs uptake (DiR signal) and mRNA translation (eGFP expression).

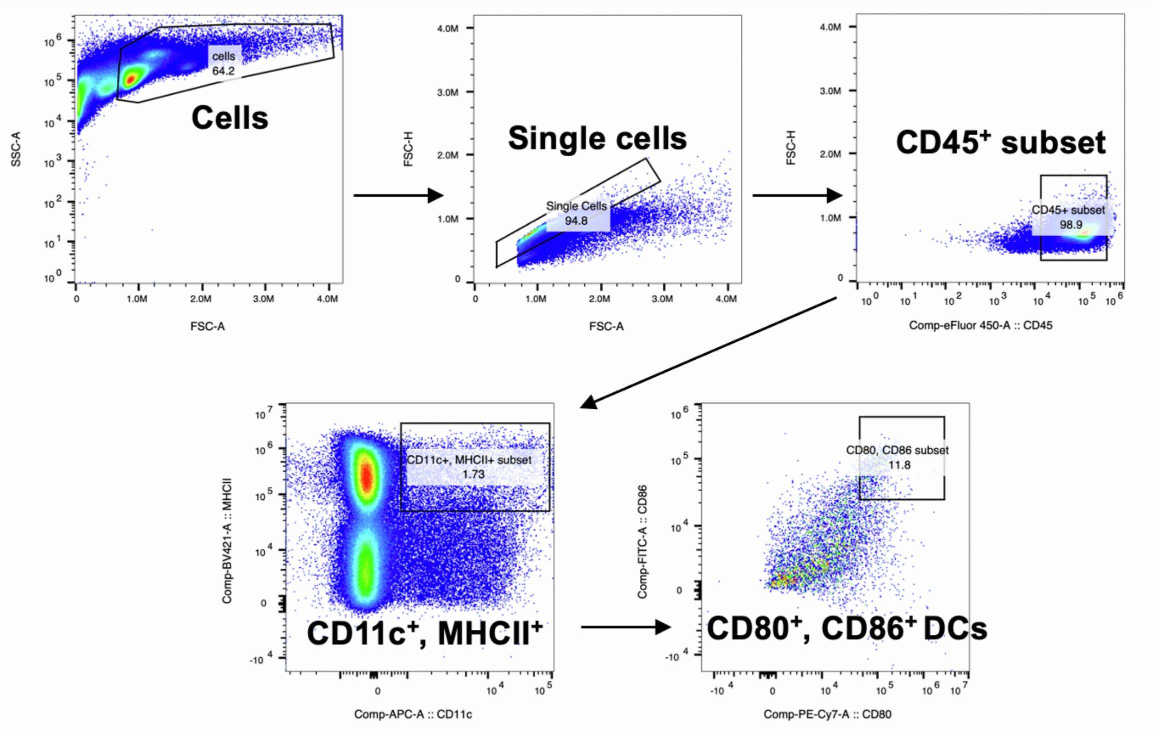

Figure S4. Flow cytometry gating strategy for identifying activated DCs ($CD80^{+}CD86^{+}$ DCs) in mouse spleens following intravenous administration of OVA-mRNA-loaded LNPs.

Splenocytes were first gated on FSC-A vs. SSC-H to exclude debris, followed by doublet discrimination (FSC-H vs. FSC-A), and live hematopoietic cells were identified using the $CD45^{+}$ marker. DCs were defined as $CD11c^{+}MHC\text{-}II^{+}$ within the $CD45^{+}$ population. The proportion of activated DCs was then determined by co-expression of CD80 and CD86. Percentages shown in each gate represent the frequency relative to the parent population. This gating scheme was used to assess DC maturation and immune activation induced by different LNPs formulations.

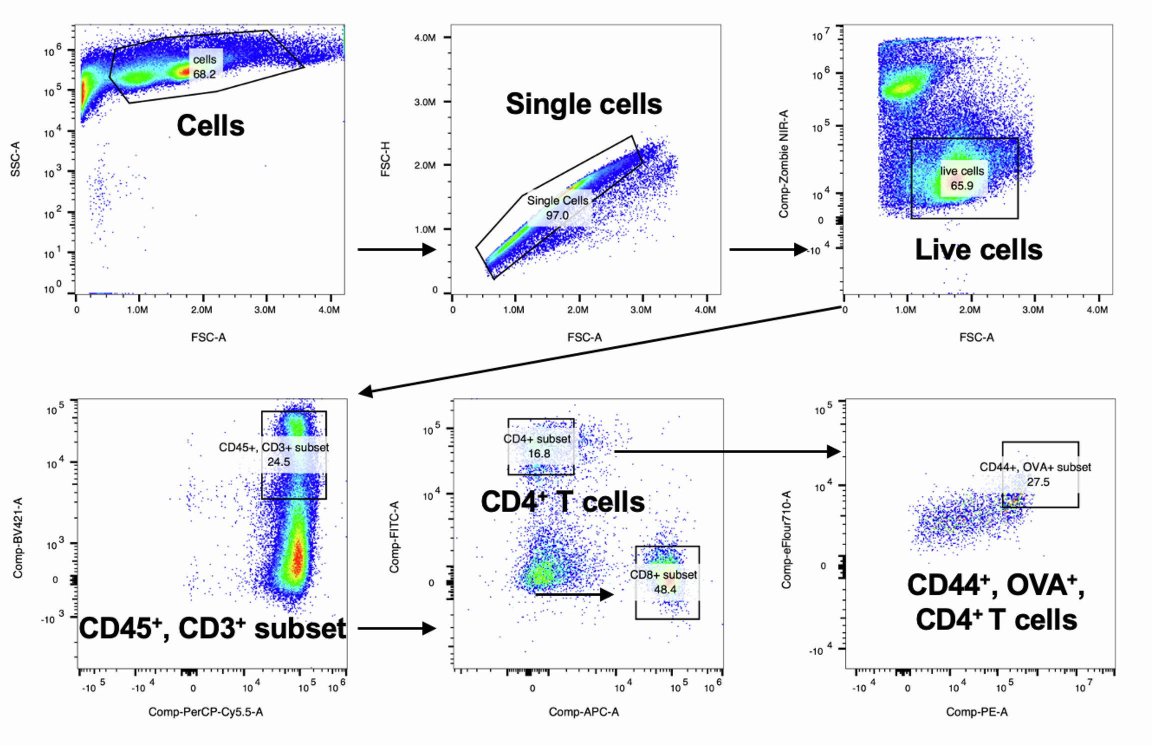

Figure S5. Flow cytometry gating strategy for identifying antigen-specific memory CD4⁺ T cells (CD44⁺OVA⁺CD4⁺) in mouse spleens following repeated intravenous administration of OVA-mRNA-loaded LNPs.

Splenocytes were first gated to remove debris and doublets, and live cells were identified as Zombie NIR⁻. T cells were selected based on CD45⁺CD3⁺ expression, followed by gating on CD4⁺ and CD8⁺ subsets. Within the CD4⁺ T cell population, cells co-expressing CD44 and SIINFEKL–MHC II tetramer (OVA⁺) were identified to quantify antigen-experienced T cells. This gating strategy was used to evaluate the magnitude of OVA-specific T cell responses induced by the different LNPs formulations.

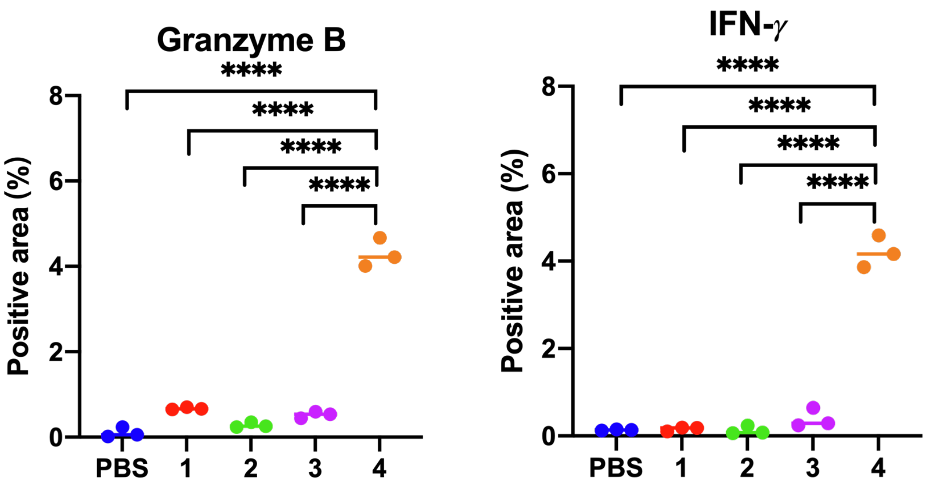

Figure S6. Semi-quantitative analysis of Granzyme B and IFN-γ IHC staining in tumor tissues after three doses of LNPs injection.

Left: Positive area (%) for Granzyme B staining; (Right: Positive area (%) for IFN-γ staining. Data are presented as mean ± SD (n = X per group). Statistical significance was determined by one-way ANOVA with Tukey's multiple comparisons test. ****$p < 0.0001$.

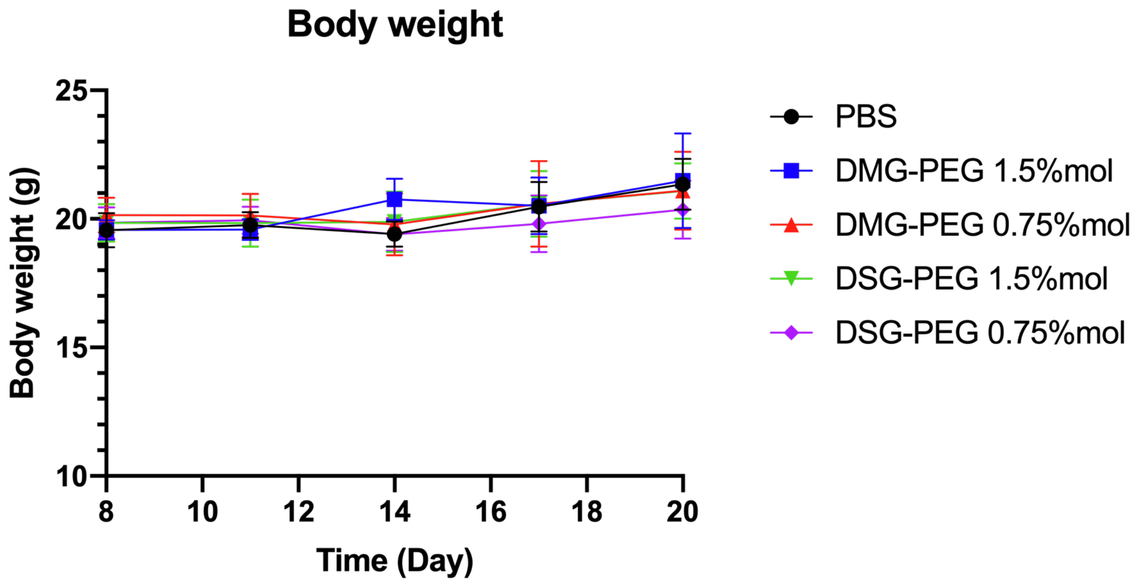

Figure S7. Body weight monitoring following three repeated intravenous injections of OVA-mRNA LNPs with different PEGylated lipid compositions.

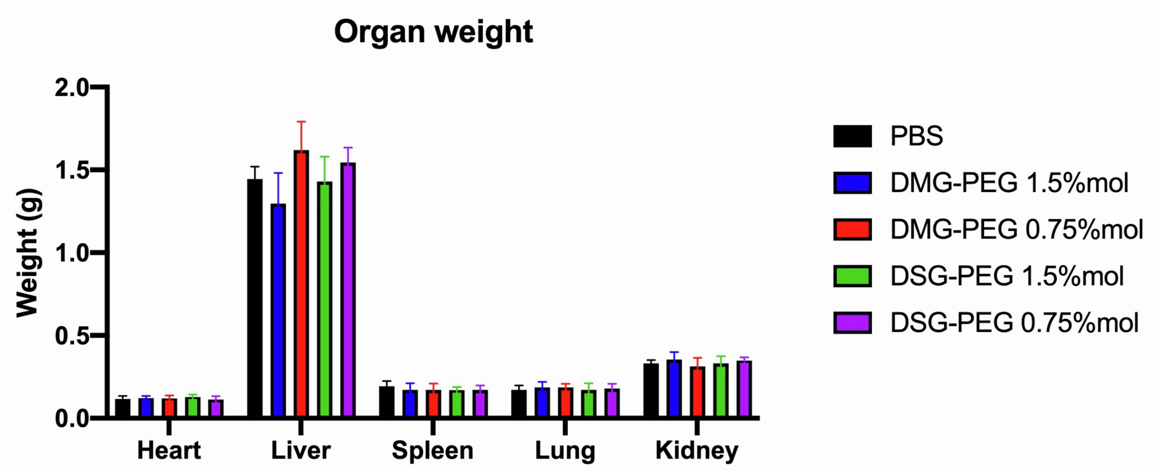

Figure S8. Organ weight analysis after three repeated intravenous injections of OVA-mRNA LNPs.

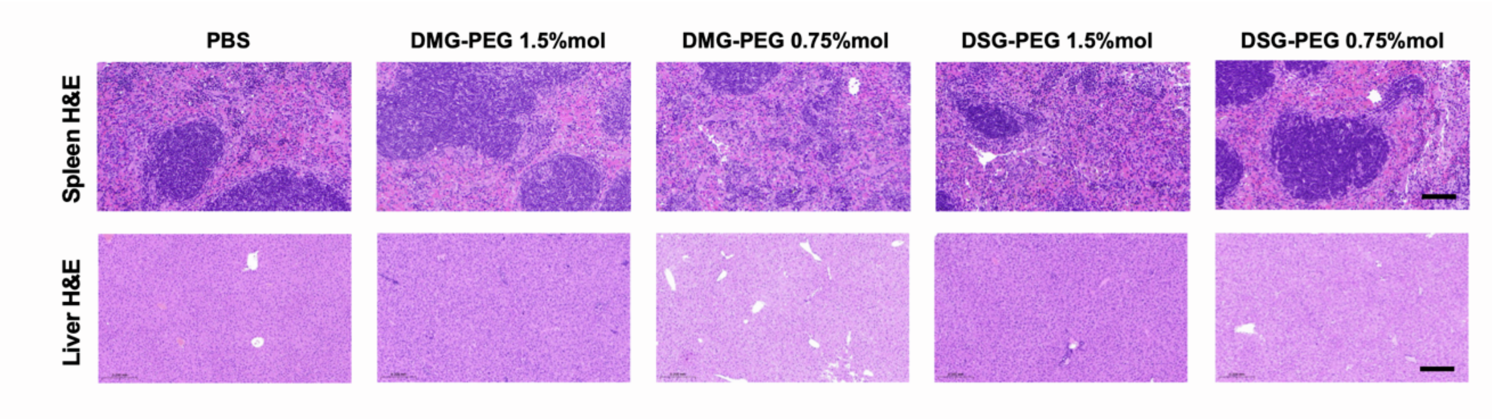

Figure S9. H&E staining for liver and spleen after three repeated intravenous injections of OVA-mRNA LNPs.

Representative H&E-stained images of spleen and liver tissues collected from mice treated with PBS, DMG-PEG (1.5 % mol and 0.75% mol), and DSG-PEG (1.5 % mol and 0.75 % mol) LNPs formulations. All groups exhibited normal splenic architecture with clearly defined white pulp and red pulp regions, and no evidence of tissue damage, necrosis, or inflammatory cell infiltration was observed. Scale bars: 200 µm.

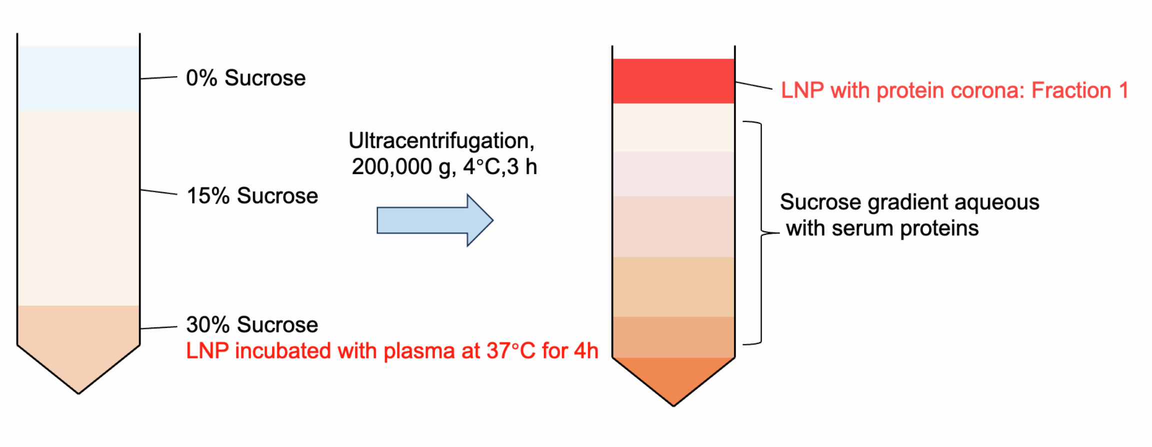

Figure S10. Schematic illustration of protein corona isolation by sucrose density gradient ultracentrifugation.

LNPs were incubated with mouse plasma at 37 °C for 4 hours to allow protein corona formation. The LNP–plasma mixture was then loaded onto the bottom of a pre-formed discontinuous sucrose gradient (30 %, 15 %, and 0 %) and centrifuged at 200,000 × g for 3 hours at 4 °C. Post-centrifugation, the sample was fractionated from top to bottom. The LNPs associated with protein corona were enriched in the top layer (Fraction 1), while the lower fractions mainly contained free serum proteins. These fractions were collected for downstream SDS-PAGE and proteomic analysis.
